# Lung function in adult patients with osteogenesis imperfecta: a cohort study

**DOI:** 10.1186/s13023-024-03452-y

**Published:** 2024-12-04

**Authors:** Alexandra Lenoir, Bérengère Aubry-Rozier, Aline Bregou, Elena Gonzalez Rodriguez, Célia Paquier, Joëlle Tanniger, Mohamed Faouzi, Romain Lazor

**Affiliations:** 1 Department of Medicine V, LMU University Hospital, LMU, Munich, Germany; 2 Department of Genetic Medicine, Lausanne University Hospital and University of Lausanne, Lausanne, Switzerland; 3 Paediatric Orthopaedic Unit, Paediatric Surgery Service, Lausanne University Hospital and University of Lausanne, Lausanne, Switzerland; 4 Interdisciplinary Centre for Bone Diseases, Rheumatology Service, Lausanne University Hospital and University of Lausanne, Lausanne, Switzerland; 5 Division of Physiotherapy, Lausanne University Hospital, Lausanne, Switzerland; 6 Division of Biostatistics, Centre for Primary Care and Public Health (Unisanté), University of Lausanne, Lausanne, Switzerland; 7 Respiratory Medicine Department, Lausanne University Hospital and University of Lausanne, Lausanne, Switzerland; 8 Service de Pneumologie, BU44.07.2137, Centre Hospitalier Universitaire Vaudois, Rue du Bugnon 46, 1011 Lausanne, Switzerland

**Keywords:** Osteogenesis imperfecta, Lung diseases/physiopathology, Spirometry, Cohort studies

## Abstract

**Background:**

Osteogenesis imperfecta (OI) is a rare hereditary bone disease resulting from a defect in collagen synthesis or processing, leading to bone fragility, frequent fractures and skeletal deformities. OI is associated with increased respiratory morbidity and mortality, but the mechanisms of lung involvement are poorly understood, and there are no data on the natural history of lung function. We studied lung function over time in a cohort of adult OI patients at one center.

**Methods:**

We used data from OI patients aged 15 and above followed up at the Lausanne university hospital between 2012 and 2023 with available pre-bronchodilator spirometry. Associations between spirometric measurements at first visit and clinical characteristics were studied through linear regression. Changes of spirometric variables over time were analysed through mixed linear regression. Models were adjusted for age, sex, height and OI type (Sillence classification).

**Results:**

Among 46 subjects, 24% had impaired spirometry at baseline, with similar distribution between restrictive (8.7%), obstructive (8.7%) and mixed (6.5%) ventilatory patterns. At first visit, higher age was associated with lower FEV_1_ (β = −0.019 l, *p* = 0.014) and lower FEV_1_/FVC (β = −0.175%, *p* = 0.012). A history of asthma was associated with higher FEV_1_ (β = 0.636 l, *p* = 0.028) and FVC (β = 0.834 l, *p* = 0.010). At first visit, FEV_1_ (β = −0.750 l, *p* = 0.006) and FVC (β = −0.859 l, *p* = 0.004) was lower in individuals with OI Sillence types 3, 4 or 5 compared to type 1. Over a mean follow-up of 3.4 years, smokers had a greater decline of FEV_1_/FVC compared to non-smokers (β = −6.592%, *p* = 0.007). Individuals with a mutation in the gene COL1A2 had 740 ml lower FVC compared to those with a mutation in COL1A1 (*p* = 0.037). After adjustment for sex, age, height and OI type, FEV_1_ increased by 26 ml (95% CI 8; 45) or 1.28%pred (0.51; 2.05) and FVC increased by 25 ml (95% CI 8; 43) or 0.93%pred (0.31; 1.55) per year of follow-up.

**Conclusions:**

An increase of FEV_1_ and FVC over time was observed in OI patients after adjustment for other variables, suggesting that the defective collagen synthesis may impact the pulmonary interstitium and lead to increased lung compliance and hyperinflation, in contrast to skeletal deformities, which reduce the thoracic volume. Lung function changes in OI thus result from the interplay of several mechanisms.

**Supplementary Information:**

The online version contains supplementary material available at 10.1186/s13023-024-03452-y.

## Introduction

Osteogenesis imperfecta (OI) is a rare hereditary disorder characterised by increased bone fragility, recurrent fractures and skeletal deformities [1, 2], with an incidence of approximately 1 in 10,000 live births [3, 4]. OI is a genetically heterogeneous disease not only affecting bones but connective tissue in general. The classic autosomal dominant form affects predominantly collagen synthesis through the COL1A1 and COL1A2 genes coding for α1(I) and α2(I) chains of collagen type I. In addition, new mainly autosomal recessive forms have been discovered in the last 15 years [5], affecting post-translational collagen changes, folding and cross-linking, as well as bone mineralization and osteoblast differentiation. This has led to the emergence of a functional genetic classification, completing the clinical modified Sillence classification introduced in 2010 by the Nosology Group of the International Skeletal Dysplasia Society, which is usually employed in clinical practice [1, 6, 7]. Sillence described initially 4 types, mainly based on clinical, radiological and mode of inheritance observations. Type 2 is a pre- or perinatal lethal form. Type 1 is a usually less severe non deforming form, with blue sclerae. Type 3 is a very severe progressively deforming form with fractures at a very early stage. Type 4 is a moderate form with generally normal sclerae. Type 5 has been introduced in 2015 in the revised International Nomenclature group for Constitutional Disorders of the Skeleton (INCDS) classification as a radiological phenotype distinguishable from type 1–4. It is outstanding that despite the multiplicity of new genes discovered, it is impossible to stick to a close correlation between genetic molecular basis and Sillence OI type 1–5. Therefore, the 2023 revision of nosology of genetic skeletal disorders still uses the Sillence phenotypical type 1–5 classification for the description of OI type [5].

Respiratory morbidity and mortality are increased in OI [8], but their prevalence and mechanisms remain poorly understood. Studies on lung function in OI patients are scarce and usually limited by small sample size [9–14]. Some authors have found a correlation between the degree of scoliosis and decreased lung function [11, 12, 15] but there are suggestions that spine deformity alone does not explain the extent of lung function abnormalities [2] and that the underlying collagen defect might play a role through structural changes affecting the lung parenchyma [14]. The largest study to date on lung function in OI patients (n = 217) showed that, for Sillence types 1 and 4, forced expiratory volume in one second (FEV_1_) and forced vital capacity (FVC) follow similar patterns compared to the general population: lung function increase in childhood and early adulthood, followed by a plateau and progressive decline from the age of 30 approximately [16]. Yet, lung volumes seem generally lower than in the general population and are particularly impacted in Sillence type 3.

Longitudinal studies on lung function changes over time have not been reported to date in individuals with OI. We aimed to describe lung function at first visit and during follow-up in adult OI patients followed at one centre.

## Methods

### Study design and study population

We analysed lung function data from OI patients aged 15 years and older followed-up prospectively at the Lausanne university hospital between 2012 and 2023 who had spirometry performed routinely as part of an annual disease evaluation [17]. Patients originated from the entire French-speaking area of Switzerland and were followed in an interdisciplinary outpatient OI clinic. The diagnosis was made by an interdisciplinary team based on clinical and radiological features, family history, and the genetic analysis; Sillence phenotypes 1–5 classification was used as discussed earlier [18]. Clinical characteristics collected at the time of the first lung function measurement included height, weight, body mass index (BMI), smoking history, history of asthma, and treatments for bone frailty (bisphosphonates, denosumab). Measurements of arm-span were not performed systematically.

### Data collection and lung function measurements

Pre-bronchodilator spirometry was performed with an EasyOne spirometer and results for FEV_1_ and for FVC were interpreted using Global Lung Function Initiative (GLI) 2012 references [19]. Obstruction was defined as the ratio FEV_1_/FVC < lower limit of normal (LLN), and restrictive pattern as FVC < LLN, where LLN refers to the 5th percentile of a healthy non-smoking population and lower values are considered abnormal. Each participant performed up to 3 spirometric manoeuvres to provide at least one acceptable blow according to European Respiratory Society/American Thoracic Society (ERS/ATS) quality standards [20]. For this study, the highest value among the acceptable blows was chosen for FEV_1_ and for FVC, even if resulting from different manoeuvres.

### Statistical analysis

Data for clinical characteristics at first visit were summarized by absolute values [range] and proportions for categorical variables, and by means and standard deviations (SD) for continuous variables. Normal distribution was checked graphically through histograms. A log transformation was performed for non-normally distributed variables.

For the variable *smoking status*, never-smokers were used as reference and current smokers and former smokers were grouped into one category. For the variable *bisphosphonate/denosumab*, denosumab only and combined bisphosphonate and denosumab treatments were grouped into one category.

Associations between lung function parameters at first visit and clinical characteristics were analysed though linear regression and, if the spirometry lung function variable was not distributed normally, through robust linear regression. Lung function changes over time were modelled through mixed linear regression for FEV_1_ and FVC. First, univariate analyses were performed to study the associations between lung function changes and the variables age, sex, height and OI type (Sillence classification). Then, a multivariate model was fitted adjusting for these variables as a priori confounders.

All analyses were conducted using STATA version 16.0 (StataCorp. 2019. Stata Statistical Software: Release 16. College Station, TX: StataCorp LLC). A significance level of < 0.05 was used.

## Results

### Clinical characteristics

Since the implementation of the OI interdisciplinary care consultation in 2012, 87 patients have been evaluated at the consultation, including 22 children. Five patients died (1 child, 4 adults) and one has been lost to follow-up to date. Five children are now adults. 56 patients were identified to be included in the present study. Among those over 15 years-old, 6 patients had no lung function evaluation, 3 had no informed consent and one was excluded from the cohort after diagnosis of a different type of connective tissue disorder. The remaining 46 OI patients with one or more lung function measurements were included, and were aged between 15 and 82 years. One patient was diagnosed with low density lipoprotein receptor-related protein 5 (LRP5) mutation, one with a type 5 OI with interferon-induced transmembrane protein 5 (IFITM5) mutation confirmed. Eight patients had a type 3 OI with a glycine substitution in six COL1A2 mutation patients, a proline substitution in one COL1A1 mutation patient, and one patient did not undergo genetic testing. Three patients had a type 4 OI, one with a recessive type X-linked plastin 3 (PLS3) mutation, one with a wingless-related integration site (WNT) mutation, and one without a genotype confirmation of COL1 mutation nor other genes in the panel. The others had type 1 OI, 21 with a dominant mutation in COL1A1, 5 with a dominant mutation in COL1A2, 5 without a genotype confirmation of COL1 mutation nor other genes in the panel, and one did not undergo genetic testing. Most patients experienced multiples fractures at a young age.

Table 1 summarizes their clinical and lung function characteristics at first visit; there was no missing data for the variables described. As OI Sillence type 1 was by far the most frequent (69.6%), it was used in the further analysis as reference whilst types 3, 4 and 5 were grouped into one category. For two patients, no Sillence type had been recorded. COL1A1 and COL1A2 were the most frequently affected genes. More than half of participants had received bone-specific treatment.

**Table 1:** Patients characteristics at first visit (n = 46)

Characteristics	n (%)
Female (%)	28 (60.9)
Caucasian ethnicity (%)	45 (97.8)
OI Sillence phenotype (%)	
1	32 (69.6)
3	8 (17.4)
4	3 (6.5)
5	1 (2.2)
Not known	2 (4.4)
Affected gene	
COL1A1	22 (47.8)
COL1A2	11 (19.6)
CREB3L1	1 (2.2)
IFITM5	1 (2.2)
LRP5	1 (2.2)
PLS3	1 (2.2)
WNT1	1 (2.2)
Unknown	8 (21.7)
Age, mean ± SD (years)	41.4 ± 16.2
Height, mean ± SD (cm)	155.5 ± 18.9
BMI, mean ± SD (kg/m^2^)	25.0 ± 6.3
Never smokers (%)	30 (65.2)
History of asthma (%)	11 (23.9)
Treatment (%)	
Biphosphonate	21 (47.7)
Denosumab	2 (4.6)
Biphosphonate and denosumab	2 (4.6)

OI: Osteogenesis imperfecta. SD: Standard deviation. BMI: Body mass index. For all described characteristics, there was no missing data

Subjects with Sillence type 1 did not differ from other Sillence types with regard to age (*p* = 0.958) but were on average 22.2 cm taller (*p* = 0.007) and 11.6 kg heavier (*p* = 0.040).

### Lung function at first visit

Table 2 shows spirometry results at first visit. Most participants had normal lung function parameters according to GLI 2012 references [19]. Four patients (8.7%) had pure restrictive pattern at baseline (FVC < LLN), four (8.7%) had pure bronchial obstruction (FEV_1_/FVC < LLN) and three (6.5%) a mixed pattern (FVC < LLN and FEV_1_/FVC < LLN). FEV_1_ and FVC were significantly higher in OI type 1 compared to other OI types (*p* = 0.004 and *p* = 0.008 respectively) whilst there was no difference in the FEV_1_/FVC ratio between OI types (*p* = 0.493).

**Table 2:** Lung function at first visit

Lung function parameter	Patient group	Absolute value (l) Mean ± SD	% predicted Mean ± SD	Z-score ± SD
FEV_1_	All patients	2.48 ± 0.85	86.7 ± 19.0	−0.97 ± 1.41
	OI type 1	2.65 ± 0.77	86.9 ± 16.6	−0.96 ± 1.21
	Other OI types	1.90 ± 0.72	87.1 ± 26.2	−0.94 ± 1.98
FVC	All patients	3.13 ± 0.96	91.4 ± 16.9	−0.65 ± 1.31
	OI type 1	3.29 ± 0.80	89.8 ± 12.8	−0.75 ± 0.97
	Other OI types	2.43 ± 0.91	94.6 ± 26.1	−0.44 ± 2.07
FEV_1_/FVC, %	All patients	78.51 ± 11.07	94.41 ± 12.71	−0.57 ± 1.34
	OI type 1	79.48 ± 10.55	96.07 ± 12.32	−0.38 ± 1.27
	Other OI types	77.64 ± 12.09	91.63 ± 12.91	−0.90 ± 1.40

FEV_1_: Forced expiratory volume in one second. FVC: Forced vital capacity. l: litres. SD: Standard deviation

Table 3 shows crude associations between spirometry parameters at first visit and main clinical characteristics. FEV_1_ was slightly lower by 19 ml with every year of age (*p* = 0.014) whilst the 14 ml lower value in FVC did not reach statistical significance. FEV_1_/FVC was slightly lower by 0.18% per year of age (*p* = 0.012). FVC was 656 ml higher in men compared with women (*p* = 0.021), and FEV_1_ was also 449 ml higher in men, although not reaching statistical significance (*p* = 0.079). FEV_1_ and FVC were both higher with increasing height (31 and 38 ml per cm respectively, *p* < 0.0001 for both) and weight (31 and 37 ml per kilogram respectively, *p* < 0.0001 for both). In participants with a history of asthma, FEV_1_ was 636 ml higher (*p* = 0.028) and FVC 834 ml higher (*p* = 0.010) as compared to individuals without asthma. FEV_1_ and FVC were respectively 750 ml and 859 ml lower in individuals with OI Sillence type 3, 4 or 5, as compared to Sillence type 1. No significant differences in lung function by smoking status were observed.

**Table 3:** Univariate associations between clinical characteristics at first visit and the spirometry parameters FEV_1_ (l), FVC (l) and FEV_1_/FVC (%) measured at first visit

Variable	FEV_1_ (l)		FVC (l)		FEV_1_/FVC (%)	
	β	*p*	β	*p*	β	*p*
Age at first visit, year	**−0.019**	**0.014**	−0.014	0.093	**−0.175**	**0.012**
Sex, male*	0.449	0.079	**0.656**	**0.021**	−3.144	0.155
Height at first visit, cm	**0.031**	** < 0.001**	**0.038**	** < 0.001**	−0.037	0.519
Weight at first visit, kg	**0.031**	** < 0.001**	**0.037**	** < 0.001**	−0.009	0.883
BMI at first visit, kg/m^2^	−0.006	0.733	−0.013	0.542	0.126	0.436
Smoking status^$^	−0.228	0.390	−0.066	0.826	−2.232	0.316
History of asthma^£^	**0.636**	**0.028**	**0.834**	**0.010**	1.752	0.443
OI type^#^	**−0.750**	**0.006**	**−0.859**	**0.004**	−1.175	0.620
Affected gene**						
COL1A2	−0.403	0.246	−0.636	0.079	−0.539	0.836
Others	0.429	0.322	0.806	0.076	−6.599	0.050
Biphosphonate/denosumab^‡^	−0.178	0.504	−0.195	0.512	−2.270	0.191

FEV_1_: forced expiratory volume in the first second. FVC: forced vital capacity. *Sex: reference is female. ^$^Smoking status: reference is never-smoker. ^£^History of asthma: reference is no history of asthma. ^#^OI type: reference is Sillence type 1 whilst types 3, 4 and 5 were grouped. **Affected gene: reference is COL1A1; in “others” were grouped CREB3L1, IFITM5, LRP5, PLS3, WNT1 and *unknown*. ^‡^Treatment with one or both (reference: neither). Linear regression used for FEV_1_ and FVC, robust linear regression used for FEV_1_/FVC (no normal distribution). Significant differences appear in bold

### Lung function change over time

The mean (SD) number of visits for all 46 participants was 2.9 (1.7), and the maximum number of visits was 8. Two or more spirometry recordings were available in 36 patients with a mean (SD) follow-up time of 3.4 (2.9) years for these individuals. Overall, height decreased by 0.16 cm (−0.27; −0.05) per year of follow-up (*p* = 0.005). Four subjects were younger than 20 years at first visit (14.9, 17.1, 18.3, and 19.8 years respectively).

There was no difference in age between Sillence type 1 and other Sillence types. Other Sillence types were on average 22 cm smaller (*p* < 0.0001) and 11 kg lighter (*p* = 0.027) compared to Sillence type 1 over the follow-up.

Table 4 shows crude associations between spirometry parameters over time and main clinical characteristics at first visit. Each additional year of age at first visit was associated with a 12 ml lower FEV_1_ (*p* = 0.030). Each additional centimetre of height at first visit was associated with a 30 ml higher FEV_1_ (*p* < 0.0001). Likewise, every kilogram of weight at first visit was associated with a 13 ml higher FEV_1_ (*p* = 0.017). Conversely, FEV_1_ was lower by 29 ml for every additional unit of BMI at first visit (*p* = 0.021). FVC was higher by 38 ml for every additional centimetre of height at first visit (*p* < 0.001) and by 18 ml for every kilogram of weight at first visit (*p* = 0.001). FVC was lower by 24 ml for every additional unit of BMI at first visit, but this was not statistically significant. Men had on average 659 ml higher FVC (*p* = 0.017) and 436 ml higher FEV_1_ (NS) compared to women. FEV_1_ was on average 808 ml higher and FVC on average 944 ml higher in individuals with OI Sillence type I (both *p* = 0.001) compared with participants belonging to the other Sillence types. FVC was on average 740 ml lower in individuals with a mutation in the gene COL1A2 compared to those with a mutation in COL1A1 (*p* = 0.037). Lower FEV_1_/FVC was significantly associated with older age at first visit (−0.20% per year, *p* = 0.005) and positive smoking history (−6.59%, *p* = 0.007). As the ratio FEV_1_/FVC was not normally distributed, Table 4 also shows results for the natural log transformed (ln) ratio FEV_1_/FVC, disclosing similar associations with age and smoking status but no other variables.

**Table 4:** Univariate linear mixed model describing the associations between clinical characteristics and change over time of spirometry parameters FEV_1_ (l), FVC (l) and FEV_1_/FVC (%)

Variable	FEV_1_ (l)		FVC (l)		FEV_1_/FVC (%)		ln (FEV_1_/FVC (%))	
	β	*p*	β	*p*	β	*p*	β	*p*
Age at first visit, year	**−0.012**	**0.030**	−0.009	0.125	**−0.203**	**0.005**	**0.014**	** < 0.0001**
Sex*	0.436	0.075	**0.659**	**0.017**	−3.008	0.256	0.240	0.097
Height at first visit, cm	**0.030**	** < 0.0001**	**0.038**	** < 0.0001**	0.031	0.658	0.002	0.656
Weight at first visit, kg	**0.013**	**0.017**	**0.018**	**0.001**	0.028	0.719	0.001	0.965
BMI at first visit, kg/m^2^	**−0.029**	**0.021**	−0.024	0.067	0.094	0.638	−0.013	0.240
Smoking status^$^	−0.171	0.342	−0.090	0.626	**−6.592**	**0.007**	**0.357**	**0.008**
History of asthma^£^	0.346	0.088	0.390	0.062	−0.859	0.771	0.047	0.771
OI type^#^	**−0.808**	**0.001**	**−0.944**	**0.001**	−2.231	0.438	0.039	0.808
Affected gene**								
COL1A2	−0.508	0.119	**−0.740**	**0.037**	0.342	0.922	−0.031	0.869
Others	0.261	0.525	0.577	0.197	−5.876	0.202	0.378	0.116
Biphosphonate/denosumab^‡^	−0.079	0.407	−0.072	0.431	0.901	0.665	0.005	0.965
Years of follow-up	−0.002	0.775	−0.003	0.690	0.096	0.687	0.005	0.708

FEV_1_: forced expiratory volume in the first second. FVC: forced vital capacity. *Sex: reference is female. ^$^Smoking status: reference is never-smoker. ^£^History of asthma: reference is no history of asthma. ^#^OI type: reference is Sillence type 1 whilst types 3, 4 and 5 were grouped. **Affected gene: reference is COL1A1; in “others” were grouped CREB3L1, IFITM5, LRP5, PLS3, WNT1 and *unknown*. ^‡^Treatment with one or both (reference: neither). Significant differences appear in bold. For FEV_1_/FVC, results for both the untransformed and the natural log (ln) transformed variable are shown as it was not normally distributed

**Fig. 1:**
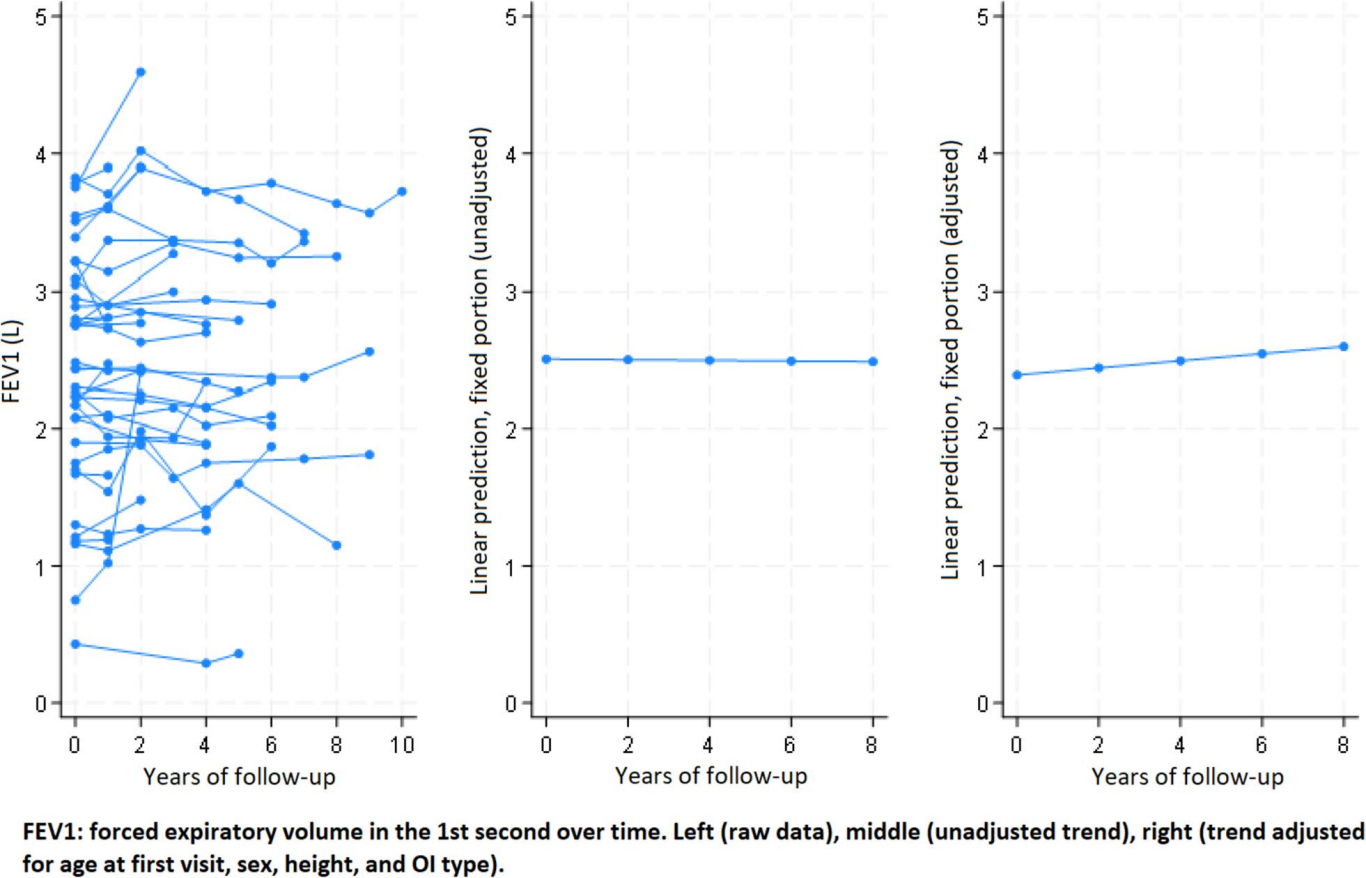
Change in FEV_1_ over time in adult patients with osteogenesis imperfecta

**Fig. 2:**
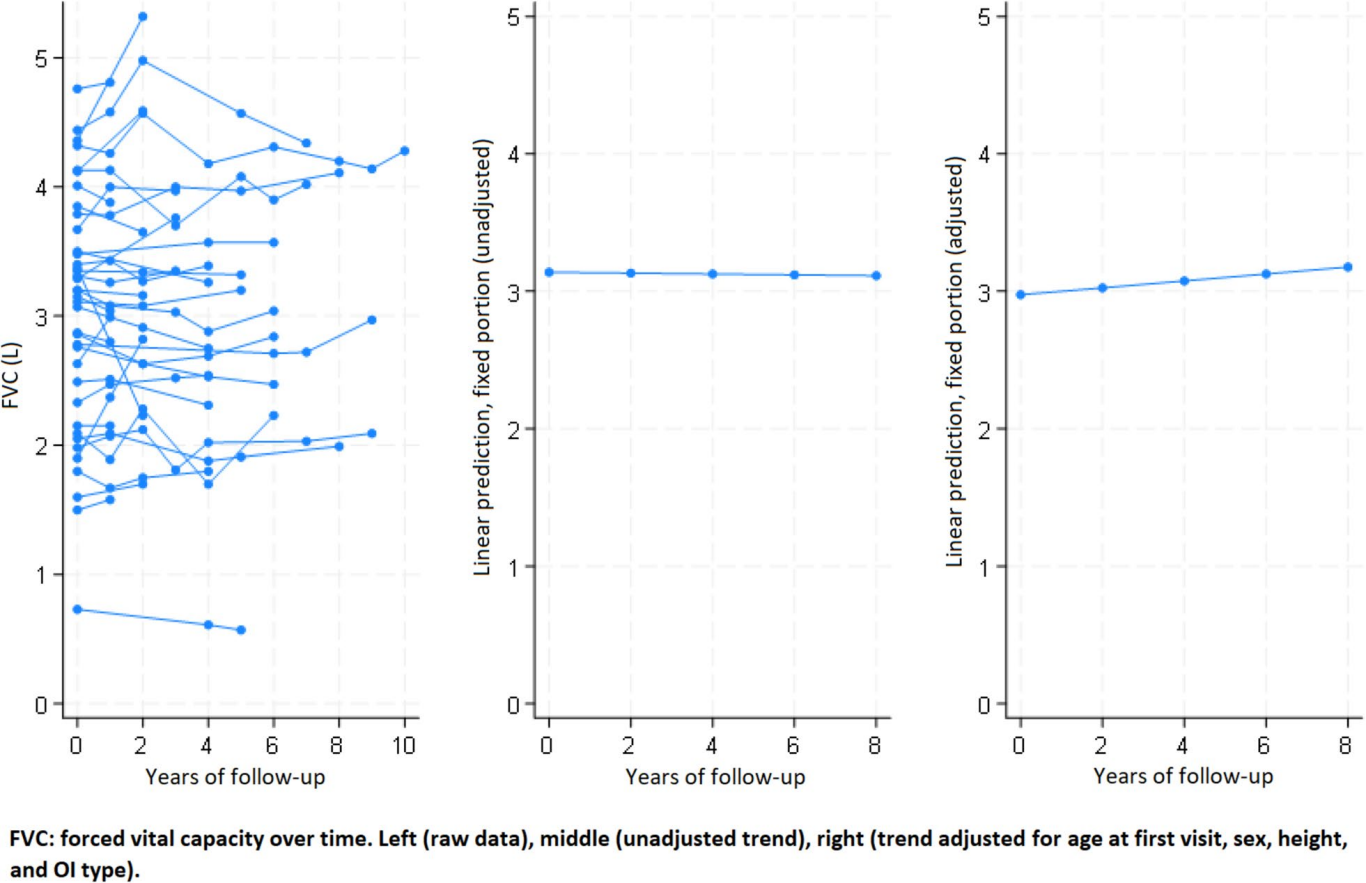
Change in FVC over time in adult patients with osteogenesis imperfecta

**Fig. 3:**
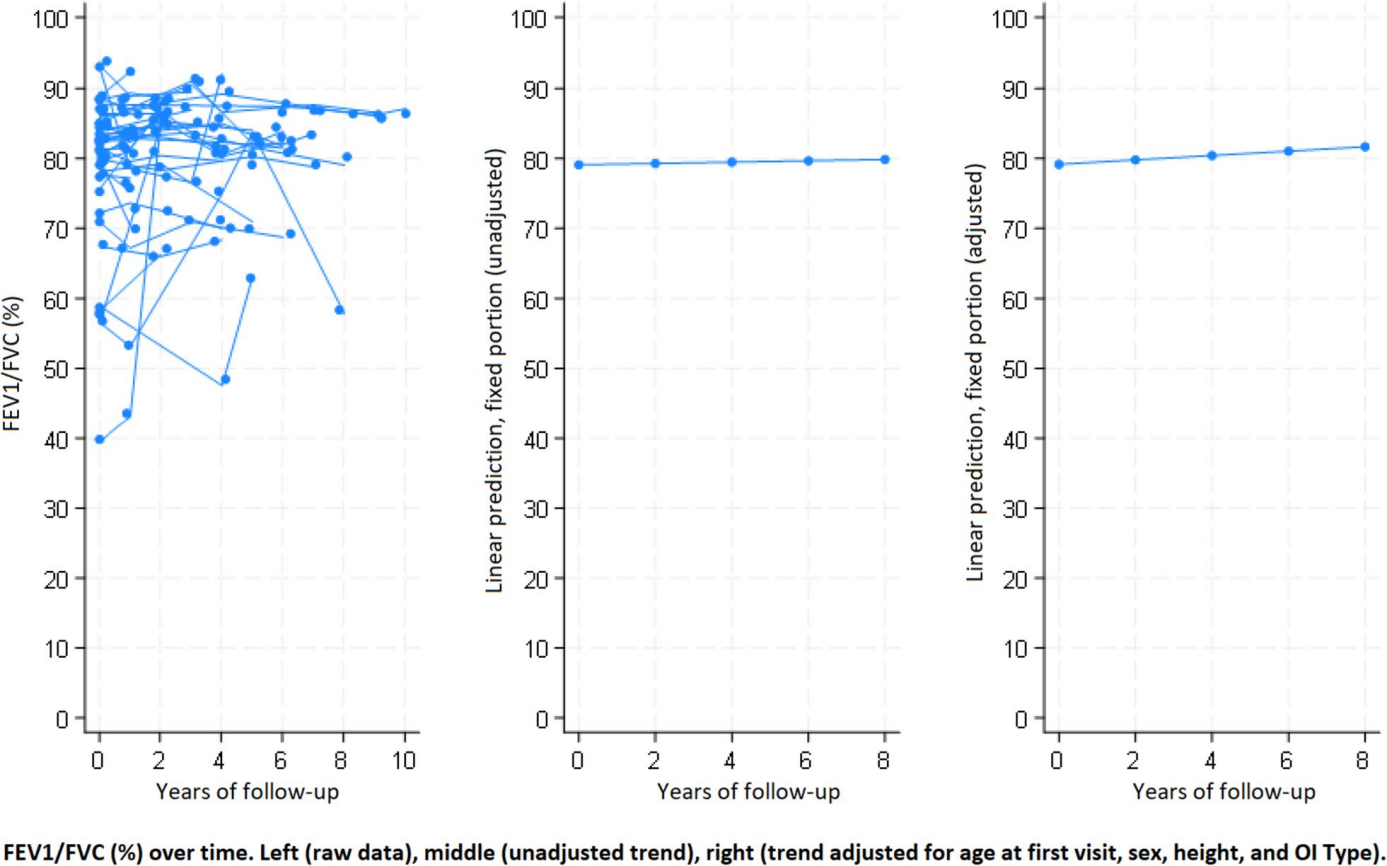
Change in FEV_1_/FVC over time in adult patients with osteogenesis imperfecta

In multivariate analysis (Table 5), FEV_1_ was lower by 23 ml (*p* < 0.0001), FVC by 21 ml (*p* < 0.001), and FEV_1_/FVC by 0.24% (*p* = 0.001) per year of older age at first visit after adjustment for sex, height, OI type and follow-up duration. FEV_1_ was higher by 28 ml and FVC by 36 ml for every additional centimetre of height at first visit adjusted for age, sex, OI type and follow-up duration. The mean changes per year of follow-up (95%CI) adjusted for sex, age, height and OI type were: FEV_1_ increased by 26 ml (8; 45) (*p* = 0.005) and FVC increased by 25 ml (8; 43) (*p* = 0.004); there was no change over time in the FEV_1_/FVC ratio in the adjusted analysis (*p* = 0.203) (Figs. 1, 2 and 3). Results for mean changes per year of follow-up for FEV_1_ and FVC in the adjusted analysis were similar when the 4 subjects aged under 20 years at first visit were excluded from the analysis (+ 24 ml, *p* = 0.012, and + 22 ml, *p* = 0.014 respectively). Expressed in percent of predicted values, the mean changes per year of follow-up adjusted for sex, age, height and OI type were: FEV_1_ increased by 1.28% (0.51; 2.05) (*p* = 0.001) and FVC increased by 0.93% (0.31; 1.55) (*p* = 0.003) (Table 6).

**Table 5:** Multivariable linear mixed model describing the association between clinical characteristics and change over time of FEV_1_ (l), FVC (l) and FEV_1_/FVC (%)

Variable	FEV_1_ (l)		FVC (l)		FEV_1_/FVC (%)	
	β	*p*	β	*p*	β	*p*
Age at first visit, year	**−0.023**	** < 0.0001**	**−0.021**	** < 0.0001**	**−0.235**	**0.001**
Sex*	0.231	0.118	**0.333**	**0.028**	−2.194	0.372
Height at first visit, cm	**0.028**	** < 0.0001**	**0.036**	** < 0.0001**	0.069	0.359
OI type^#^	−0.197	0.287	−0.168	0.375	−0.440	0.887
Years of follow-up	**0.026**	**0.005**	**0.025**	**0.004**	0.314	0.203

FEV_1_: forced expiratory volume in the first second. FVC: forced vital capacity. *Sex: reference is female. ^#^OI type: reference is Sillence type 1 whilst types 3, 4 and 5 were grouped. Results in **bold** show significant differences according to the conventional threshold of p < 0.05

**Table 6:** Multivariable linear mixed model describing the association between clinical characteristics and change over time of FEV_1_ (%predicted), FVC (%predicted) and FEV_1_/FVC (%predicted)

*Variable*	FEV_1_ (%pred)		FVC (%pred)		FEV_1_/FVC (%pred)	
	β	*p*	β	*p*	β	*p*
Age at first visit, years	−0.273	0.116	−0.241	0.099	−0.058	0.508
Sex*	−9.692	0.104	−8.846	0.078	−1.551	0.601
Height at first visit, cm	−0.047	0.793	−0.167	0.267	0.171	0.060
OI type^#^	−1.467	0.844	−0.670	0.915	−0.965	0.795
Years of follow-up	**1.277**	**0.001**	**0.932**	**0.003**	0.414	0.166

FEV_1_: forced expiratory volume in the first second. FVC: forced vital capacity. *Sex: reference is female. ^#^OI type: reference is Sillence type 1 whilst types 3, 4 and 5 were grouped. Results in bold show significant differences according to the conventional threshold of p < 0.05

Supplementary Tables S1 and S2 show multivariate analyses of the absolute and percent predicted lung function values after natural log (ln) transformation, which was performed because the FEV_1_/FVC ratio did not fit a normal distribution. Results confirmed those of Tables 5 and 6 regarding the significant change of FEV_1_ and FVC with years of follow-up. Supplementary Table S3 shows the results of the multivariable analysis expressed as Z-scores, which correspond to the %predicted in Table 6 and confirm its results.

## Discussion

To our knowledge, this study is the first to provide prospective longitudinal data through multivariate analysis on lung function in patients with OI. At first visit, 24% of studied individuals had impaired spirometry, with a roughly equal distribution between restrictive (n = 4), obstructive (n = 4) and mixed patterns (n = 3). Our main finding was that, over a mean follow-up duration of 3.4 years, FEV_1_ increased over time by 26 ml per year and FVC increased by 25 ml per year after adjustments for age, sex, height and OI type. To our knowledge, this is the first observation of an increase in lung volumes over time in patients with OI. This finding appears contra-intuitive, as a decrease due to skeletal deformities would rather have been expected. It sheds new light on the mechanisms which could affect lung function in OI.

Studies on lung function in OI patients have been generally sparse [10–13, 16, 21–26]. The majority has reported decreased lung function, which was attributed to extra-pulmonary abnormalities, in particular spinal deformities but also pectus carinatum and brittle ribs. In these studies, restrictive pattern was the predominant lung function abnormality, found in 15% [10] to 88% [24] of the studied population. In the present study, a restrictive or mixed (both restrictive and obstructive) pattern was found in 7/46 (15%) of our patients at first visit, i.e. similar to some previous reports with a rather low prevalence. Methodological issues could explain this variability. Indeed, as measurement of total lung capacity was usually not available, restrictive pattern was often defined as FEV_1_/FVC > 80% in other studies [11, 24, 26], a rather rough criterion. In the present study, we used FVC < LLN to define restrictive ventilatory pattern, which we believe is more precise as it uses available recent reference equations rather than relying on a ratio [27] and might therefore explain the smaller prevalence of restrictive pattern in our study. Another possible explanation could be the high proportion of Sillence type 1 patients in our cohort (73%), which usually corresponds to a rather mild phenotypic expression of the disease. Obstructive ventilatory pattern was found in a minority of our patients, similar to the findings of Gochuico and colleagues [23].

Our multivariate analysis shows a positive association between lung volumes and height as well as a negative association between lung function and age. This is in line with previous studies on adults with no known respiratory disease [28] and the recommendations for current reference values [19] where height and age are the most important predictors of FEV_1_ and FVC in the general population. In OI, a large multicentric cross-sectional study of 217 patients [16] disclosed higher FEV_1_ and FVC with higher age until the third decade, and a decline thereafter. In line with these findings, we found a negative association between age at first visit and both FEV_1_ and FVC in our population having a mean age at first visit of 41.4 years (Table 3).

The well-known relationship between cigarette smoking and lower lung function as well as faster lung function decline [29, 30] is also reflected in our results with a greater FEV_1_/FVC decrease over time in smokers, even though this could only be assessed in univariate analysis due to small sample size. These observations in OI patients seem therefore to correspond to the general population.

The prevalence of a history of asthma seems rather high in our cohort (24%) compared to the general population in Switzerland, where it is estimated around 4–10% [31, 32], possibly due to an increased health care use and therefore lower diagnostic threshold in the OI population. Our results show higher FEV_1_ and FVC in individuals with a history of asthma, which could be explained by use of bronchodilator medication or by a possible training effect in individuals who regularly perform spirometry.

Lung function differed between OI phenotypes, with Sillence type 1 individuals displaying significantly higher lung function than Sillence types 3, 4 and 5 combined. This is in line with the milder phenotypic expression of OI in Sillence type 1 and observations made in previous studies [10, 16, 26]. Of note, we found no difference between Sillence type 1 and types 3, 4 and 5 regarding change over time of adjusted spirometric variables (Table 5).

The most relevant and novel finding of the present study was the significant increase of lung volumes over time after adjustment for age at first visit, sex, height and OI type. The increases of FEV_1_ and FVC were of the same magnitude (26 and 25 ml per year, respectively), and contrast with the expected decrease of these parameters in healthy subjects. Indeed, FEV_1_ decreases physiologically between 18 and 46 ml per year in healthy nonsmokers, probably accelerating with age [28]. The observed increase in lung volumes is also unexpected in OI patients, as a decrease attributed to skeletal deformities has been reported previously in cross-sectional studies. Furthermore, the observed increase in lung volumes occurred despite a mean decrease in height of 0.16 cm per year of follow-up, which would also be expected to decrease lung volumes. Therefore, our observation cannot be explained by previous cross-sectional observations in OI, and no longitudinal studies are currently available for comparison. In addition, we did not find any relevant change in results when repeating the analysis without the 4 subjects younger than 20 years at first visit, thus eliminating this possible confounder. Of note, the observed increase in lung volumes over time in the same individuals is not in contradiction with the fact that lower FEV_1_ and FVC were associated with higher age at first visit across the study population.

A possible explanation for the observed increase in lung volumes over time could be an effect of OI on the lung parenchyma itself. Bronheim et al*.* and Khan et al*.* found no correlation between degree of scoliosis and pulmonary function [2, 26], suggesting that the observed changes in lung function must be explained by other factors. Indeed, several recent publications suggest that collagen defects contribute to respiratory morbidity and mortality in OI patients not only through structural changes of the thoracic cavity but also through intrinsic alterations of lung tissue [2, 14, 26]. Alongside restrictive lung function pattern in more than half of their studied cohort, Gochuico and colleagues also found on chest CT various abnormalities, most commonly small bronchial thickening but also bronchiectasis, atelectasis, reticulation, ground glass opacities and emphysema, the latter correlating with impaired diffusing capacity [23]. Chiapetta and colleagues described two giant bullae in a patient with type 4 OI [49]. Thiele et al. described pulmonary function decline in a pediatric OI cohort independently of scoliosis, as well as hemorrhage, inflammation, markers of hypoxia, hypertension and defects in tissue angiogenesis in the lung tissue of an OI mouse model [14]. Other animal experiments showed the occurrence of alveolar airspace enlargement [33, 34], as well as reduced diaphragmatic muscle mass and intrinsic contractile weakness in mouse models of OI. In one study, alterations in the mice’ respiratory mechanics and increased respiratory elastance could additionally be demonstrated alongside changes in the lung parenchyma similar to emphysema [35]. The same group published a few years later more data from an OI mouse model showing enlarged acinar airspace and reduced alveolar surface altering respiratory mechanics [36]. A few case reports provide histological information on Sillence type 2 individuals who died at birth or shortly after, showing decreased quantity of lung parenchyma, decreased alveolar number per acinus, increased volume density of alveolar and alveolar duct spaces and parenchymal septal tissue, lower proportion of respiratory bronchioles and signs of diffuse alveolar damage [37–39]. Thus, although various changes of the lung parenchyma have been observed in humans and animal models, several findings hint towards emphysematous changes in OI.

A role of the underlying collagen defect in OI has been suggested [40] because collagen in general, and collagen I in particular, are major structural elements of the lung, present in airways, blood vessels and interstitium [41]. Collagen types I and III, in a ratio of approximately 2:1, are the main fibrous components of the interstitium, representing more than 90% of all parenchymal collagens. Together with elastin, collagen is a major component of the lung connective tissue network and provides the lung with its elasticity and tensile strength [42]. Some data suggest that collagen degradation through collagenase may play an important role in the pathogenesis of emphysema [43]. In a transgenic murine model of emphysema in which pulmonary expression of collagenase transgene was used to alter the extracellular matrix, disruption of lung architecture with dilated distal airspaces, bullous lesions and lung hyperinflation were observed, suggesting that collagen is damaged in emphysema [44]. In a model of mice exposed to cigarette smoke to induce emphysema, reduced expression of COL1A1 and reduced collagen type I were observed in smoking mice as compared to controls [45]. In a rat model of emphysema induced by intratracheal instillation of elastase followed after 4 weeks by analysis of lung mechanical properties and immunostaining, emphysematous lungs were characterized by increased compliance and rupture of collagen fibers under mechanical strain, suggesting that collagen contributes with elastin to normal lung elasticity, and that emphysema is associated with damage to collagen fibers, which appear thicker but weaker than in normal lungs [46]. In Ehlers-Danlos syndromes characterized by primary defects in fibrillar collagens type I, III and V, emphysematous changes of the lung have also been observed both in humans [47] and in murine models [48]. One could therefore hypothesize that loss of collagen in OI could lead to increase in lung compliance and increase in lung volumes similarly to what is observed in emphysema [23, 35, 49]. In OI, altered and dysfunctional collagen leads to disturbed bone remodeling mechanisms and subsequently low bone mass and increased bone fragility but also to dental abnormalities, neurological abnormalities and hearing loss [50]; comparable changes in human lung tissue have not been described to date.

As an alternative explanation for the increased lung volumes observed over time in the present study, one could hypothesize that the effect of bone-specific treatment may have stabilized the thoracic cage and have attenuated the restrictive component of the lung function impairment. However, treatment with bisphosphonates and/or denosumab was not associated with lung function in our univariate analysis, although it could not be adjusted for in the multivariate model because of small sample size. In any case, it seems unlikely that a pharmacological consolidation of the thoracic cage would result in increased lung volumes, and stability would at most be expected. Likewise, is seems unlikely that OI-induced changes in muscle mass and/or contractility, as reported in animal models [33–35], would increase lung volumes, even if the patients of our cohort did improve their physical condition by regular physiotherapy and physical activity as described elsewhere [17].

Our study has limitations. First, due to disease’s rarity, our patient cohort was small, limiting the possibilities of subgroup analyses, e.g. regarding Sillence types. Second, the validity of the predicted lung function values may be questioned for our cohort, especially in individuals with Sillence type 3, 4 and 5, who usually present with more severe bone deformities than type 1 patients. Height is an essential parameter to calculate the predicted values for FEV_1_ and FVC [19], and arm span height might have been a more accurate measurement than standing height in these subjects [51] but was unfortunately not systematically measured at the time of the patients’ visits. Of note, it has been described that individuals with OI type 1 can have a normal lung function if the arm span is used to define the predicted values [9, 10]. However, the absolute values of lung function measurements are not affected by this limitation and change over time in absolute and predicted values both evolve in the same direction in our study, i.e. towards an increase in lung volumes. Therefore, our conclusions clearly remain valid despite the lack of arm span height to determine predicted values instead of standing height. Third, as no clear recommendations had been published until recently [52] regarding standard lung function testing in individuals with OI, spirometry data in our patients originated from our own initiative to offer lung function evaluation to patients on their regular check-up appointments, also in the absence of respiratory symptoms. Uptake was therefore irregular and subject to some selection bias. Study participants might have been particularly adherent to medical care and mindful of their respiratory health. Nevertheless, our data is a valuable sample of lung function measurements in patients with this rare disease. Fourth, we did not perform chest computed tomography in our patients and could therefore not perform correlations between lung function and structure. Future studies could include such data, including quantitative assessment of lung tissue density.

## Conclusion

In this first longitudinal observational study of lung function in adults with OI, we demonstrated that FEV_1_ and FVC slightly increase over time after adjustments for other variables. The pathophysiologic mechanism behind this observation could be linked to an intrinsic effect of the underlying collagen defect on the lung parenchyma. Larger cohorts with longer follow-up are needed to confirm these findings and link them to the high respiratory morbidity and mortality seen in OI patients. For this reason, but also to pick up on early changes, standardization of regular lung function measurements should be implemented for individuals with OI.

## Supplementary Information

Additional file 1.

## Data Availability

The datasets used and/or analysed during the current study are available from the corresponding author on reasonable request.
