# Supplementary material for: Lung function in adult patients with osteogenesis imperfecta: a cohort study
Source: Orphanet J Rare Dis. 2024 Dec 4;19:455. doi: 10.1186/s13023-024-03452-y (PMC11616175; doi:10.1186/s13023-024-03452-y)
Supplement: Supplementary file 1 — Additional file 1. [file 13023_2024_3452_MOESM1_ESM.docx]

**Lung function in adult patients with Osteogenesis imperfecta.**

**A cohort study**

**Supplementary information**

**Supplementary table S1:** Multivariable linear mixed model describing the association between clinical characteristics and change over time of FEV_1_ (l), FVC (l) and FEV_1_/FVC (%) after natural log (ln) transformation of the spirometric variables.

| **Variable** | **lnFEV_1_ (l)** | | **lnFVC (l)** | | **ln(FEV_1_/FVC) (%)** | |
| --- | --- | --- | --- | --- | --- | --- |
|  | **β** | **p** | **β** | **p** | **β** | **p** |
| Age at first visit, year | **0.001** | **<0.001** | **0.001** | **<0.001** | **0.015** | **<0.001** |
| Sex* | -0.011 | 0.093 | **-0.009** | **0.022** | 0.192 | 0.144 |
| Height at first visit, cm | **-0.001** | **<0.001** | **-0.001** | **<0.001** | -0.001 | 0.763 |
| OI type^#^ | 0.009 | 0.270 | 0.004 | 0.369 | -0.006 | 0.971 |
| Years of follow-up | **-0.001** | **0.006** | **-0.001** | **0.005** | -0.009 | 0.494 |

FEV_1_: forced expiratory volume in the first second. FVC: forced vital capacity. *Sex: reference is female. ^#^OI type: reference is Sillence type I whilst types III, IV and V were grouped. Results in **bold** show significant differences according to the threshold of p<0.05.

**Supplementary table S2:** Multivariable linear mixed model describing the association between clinical characteristics and change over time of FEV_1_ (%predicted), FVC (%predicted) and FEV1/FVC (%predicted) after natural log (ln) transformation of the dependent variables.

| ***Variable*** | **lnFEV_1_ (%pred)** | | **lnFVC (%pred)** | | **ln(FEV_1_/FVC) (%pred)** | |
| --- | --- | --- | --- | --- | --- | --- |
|  | **β** | **p** | **β** | **p** | **β** | **p** |
| Age at first visit, years | 0.002 | 0.156 | 0.001 | 0.112 | -0.0001 | 0.972 |
| Sex* | 0.070 | 0.171 | 0.036 | 0.108 | 0.030 | 0.848 |
| Height at first visit, cm | 0.001 | 0.419 | 0.001 | 0.181 | -0.008 | 0.102 |
| OI type^#^ | 0.008 | 0.903 | 0.001 | 0.969 | 0.095 | 0.631 |
| Years of follow-up | **-0.012** | **0.001** | **-0.004** | **0.004** | -0.015 | 0.349 |

FEV_1_: forced expiratory volume in the first second. FVC: forced vital capacity. *Sex: reference is female. ^#^OI type: reference is Sillence type I whilst types III, IV and V were grouped. Results in **bold** show significant differences according to the threshold of p<0.05.

**Supplementary table S3:** Multivariable linear mixed model describing the association between clinical characteristics and change over time of FEV_1_ (Z-score), FVC (Z-score) and FEV_1_/FVC (Z-score).

| ***Variable*** | **FEV_1_ (Z-score)** | | **FVC (Z-score)** | | **FEV_1_/FVC Z-score)** | |
| --- | --- | --- | --- | --- | --- | --- |
|  | **β** | **p** | **β** | **p** | **β** | **p** |
| Age at first visit, years | -0.014 | 0.275 | -0.013 | 0.232 | -0.004 | 0.678 |
| Sex* | 0.668 | 0.130 | 0.675 | 0.081 | 0.196 | 0.537 |
| Height at first visit, cm | -0.008 | 0.549 | -0.014 | 0.218 | 0.017 | 0.078 |
| OI type^#^ | -0.199 | 0.719 | -0.161 | 0.740 | -0.167 | 0.675 |
| Years of follow-up | **0.098** | **0.001** | **0.069** | **0.005** | 0.042 | 0.181 |

FEV_1_: forced expiratory volume in the first second. FVC: forced vital capacity. *Sex: reference is female. ^#^OI type: reference is Sillence type 1 whilst types 3, 4 and 5 were grouped. Results in **bold** show significant differences according to the conventional threshold of p<0.05.
